# The Birmingham Hospital Saturday Fund

**Published:** 1894-06-23

**Authors:** W. T. Smedley


### Editor's Letter-Box.

[Our correspondents are reminded that proxility is a great bar to publication, and that brevity of style and conciseness of statement greatly facilitate early insertion.]

### THE BIRMINGHAM HOSPITAL SATURDAY FUND.

Mr. W. T. Smedley, the Honorary Secretary, writes :—

It is with reluctance that I again refer to your articles upon the subject, but you have strayed so far from the real issues that I must ask for space to state with brevity what these are.

The discussion originated in a statement made by Mr. Burdett that the present position of the Birmingham Hospital Saturday Fund was more attributable to the foundations laid by Mr. Gamgee, assisted by Mr. George Dawson and Mr. G. F. Muntz, than to any other cause. Mr. Burdett, during his speech, mentioned no other cause as contributing even in any slight degree to the present position of the Fund, and the only construction, therefore, that any fair-minded person can place upon his words is that to the cause he mentioned, and to no other, in his opinion, is that position due. Mr. George Dawson, neither directly nor indirectly, had anything to do with the movement. The beginning and the end of Mr. Muntz's interest was a gift of £500 twenty-one years ago. The foundation of Mr. Gamgee's Hospital Saturday was "emotion"; the foundation of the success has been "organisation"—which he deprecated. You say, "Method of collection was a detail"; Mr. Gamgee says it is everything. His words are :—

The complete success of Hospital Saturday depends in no small measure upon the method of collecting the money, and this is a matter attended with no small difficulty. The projectors of Hospital Saturday did not aspire to make it a success on the mere mechanical principle of "Many a mickle makes a muckle." The penny a week system in factories and workshops is vastly older than Hospital Saturday, in instituting which it was hoped to sanctify a labour day, by the sacrifices and loving thoughts of those yet blessed with the strength to work on behalf of fellow toilers laid low by injury or disease. Leaving practical religion to work its power in soothing pain, strenuous action—that next grand remedy of human ills—was the agency on which we relied for the success of our first Hospital Saturday, March 15th. For that day the men with big muscles, the women and children with nimble fingers, gave one, two, three, and in some cases more, hours' labour; that out of the excess of their health and strength, those whom accident had deprived of the use of their limbs, those whose very life blood had been thinned or poisoned by insiduous disease, might in their helplessness be comforted by the thought that, as they were lying helpless objects of charity, the sweat was trickling down the heavy ironworkers' brow, the eyes and fingers of the skilled artisan were being strained to work for Hospital Saturday, that the blind and the palsied might have sight and strength.

The points you have picked out from a cursory inspection of Mr. Gamgee's lecture on the "Origin and Future of Hospital Saturday" were not put forward by him until ten years after the movement had passed out of his hands, and when it had achieved success and was on the high road to further triumphs.

On the free gifts question you persistently ignore the real point. The roadmen subscribed £94, spent £41 in tickets and registration fees, and had £53 left. They could divide it amongst themselves, spend it in a picnic, or purchase hospital tickets with it. They elected to give it to the Hospital Saturday Fund without asking anything in return. Was that gift a free one or was it not? That is the point at issue. [No decision as to what portion, if any, of the £53 was a free gift can be arrived at until the actual loss entailed upon the hospitals by the purchase of the £41 worth of tickets has been ascertained. Will Mr. Smedley see that this information is forthcoming in future reports of this Fund?—Ed. *T. H.*]

I am fully acquainted with the details of the Wolverhampton and Staffordshire Hospital Saturday Committee. Is Mr. Edwin A. White the secretary? Does he perform the work of the Hospital Saturday Fund as part of his duties as secretary of the hospital, receiving from the hospital a percentage on the amount raised? Is the whole of the work done by the hospital staff, and do the accounts appear as part of the hospital accounts? That is the point at issue. [Mr. Smedley now varies his ground, and is in conflict with the evidence as we have already stated.—Ed. *T. H.*]

Was Mr. Burdett's assertion accurate that the working men of Wolverhampton contributed £39 per thousand of population? Or is it a fact that the Wolverhampton contributions amount only to £22 per thousand, and that he had calculated a figure which included gifts from Wednesbury, Walsall, Dudley, Darlaston, and other towns, on the population of Wolverhampton only? Can the slightest reliance be placed on statistics of which this is a sample? You say the tickets given to the working men in Wolverhampton do not give them the right to indoor treatment. Do not ordinary subscribers receive two tickets for each guinea subscribed, giving the owner the right to indoor or outdoor treatment as may be considered most needful to his case by the hospital medical men? Workmen's subscriptions give them exactly the same privileges. [The figures referred to are worked out on the same basis for Birmingham and Wolverhampton, as supplied by the respective authorities. The tickets referred to confer no such rights as Mr. Smedley states.—Ed. *T.H.*]

What sin have we committed which should cause Mr. Burdett to go out of his way to try and make out that the Birmingham Hospital Saturday Fund is to take very low rank amongst similar institutions in the country when previously in his "Hospital Annual" he has always given it the foremost position? Having endeavoured to knock it down to the bottom of the list, why should he take hold of the little credit that remains for having kept some life in it, though, as he would contend, that life be very slight, and place it to the account of men who have had nothing whatever to do with it.

By making claims for Mr. Gamgee, which everyone who has the least knowledge of the subject knows are absolutely without foundation, you run a great risk of robbing him of the credit to which he is justly entitled as the founder of the movement in Birmingham. As to "words," you have published six columns from my pen, and your articles have filled thirteen and a half columns, so my "avalanche" is the smaller of the two.

*** We regret the spirit Mr. Smedley's letter exhibits. It is unworthy of his better self and the cause he represents. Mr. Gamgee's reputation does not rest, as Mr. Smedley implies, upon any less sure evidence than that afforded by his published utterances and life's work which negative Mr. Smedley's contentions. History will no doubt do full justice to Mr. Gamgee and his co-helpers, and it is out of the power of any one person or any body of persons to unduly belittle either one or the other. When all due credit has been given to those good men and true who are now dead, enough surely remains to satisfy the most ravenous of their successors. No claims have been advanced in these columns or elsewhere for Mr. Gamgee to which he is not fully entitled, as the evidence to which Mr. Smedley appealed fully proves. In saying this we wish once again to do full justice to the self-denying and successful labours of Mr. Alderman Cooke, Mr. Smedley, and the present members of the committee of the Hospital Saturday Fund—labours which were fully and gratefully acknowledged in our articles published in The Hospital of May 12th and 26th, 1893, which Mr. Smedley so strangely ignores. They were never, however, in question for one moment, as every fair-minded person who reads the whole controversy must readily admit. We need not, therefore, labour the point further. We hope that Mr. Smedley will now set to work, and, with the aid of his committee, not only adopt but improve upon the Potteries system by publishing each year adequate and full accounts, which will enable everybody to ascertain how far, if at all, a free gift is made to the hospitals by the Birmingham Hospital Saturday Fund.—Ed. *T. H.*

